# Prevalence of and risk factors for periodontal disease among pregnant women in an antenatal care clinic in Khartoum, Sudan

**DOI:** 10.1186/s13104-020-04998-3

**Published:** 2020-03-11

**Authors:** Yasir Salih, Abubakr M. Nasr, Abdel B. A. Ahmed, Manal E. Sharif, Ishag Adam

**Affiliations:** 1 Faculty of Medicine, University of Khartoum, PO box 102, Khartoum, Sudan; 2 College of Medicine, King Khalid University, Abha, Kingdom of Saudi Arabia

**Keywords:** Periodontal disease, Pregnancy, Risk factors, Sudan

## Abstract

**Objectives:**

The aim was to investigate the prevalence of and factors associated with periodontal disease among pregnant Sudanese women. A cross-sectional study was conducted at the Antenatal Care Clinic of Saad Abualila Hospital (Khartoum, Sudan) from August to October 2018. Socioeconomic-demographic information and reproductive history were gathered using a questionnaire. Body mass index was computed from the weight and height. The diagnosis of periodontal disease was performed using criterion that also evaluated bleeding upon probing.

**Results:**

Four hundred and four women were enrolled in the study, with a mean (SD) gestational age of 30.0 (8.7) weeks. Their mean (SD) age and parity were 27.0 (5.7) years and 1.6 (1.7), respectively. Ninety-seven (24.0%) of these 404 women had periodontal disease, which was mild, moderate and severe in 49 (12.1%), 36 (8.9%) and 12 (3.0%) women respectively, while 307 (76.0%) women had no periodontal disease. In logistic regression, age, parity, education, and brushing were not associated with periodontitis, but lower gestational age was associated with periodontal disease (OR = 0.96, 95% CI 0.94–0.99, P = 0.011).

## Introduction

Periodontal disease is an inflammatory process/condition of the soft tissues that surround the teeth characterized by gradual destruction of the soft tissue that supports the structures of the teeth [1]. Periodontal disease is an infectious disease distributed worldwide that can affect up to 90% of populations [2, 3]. Poor oral hygiene, increasing age, smoking, low educational level, some ethnicities, and poor economic status have been reported as risk factors for periodontal disease [1–4].

During pregnancy, due to hormonal factors (high oestrogen and progesterone), women are more vulnerable to periodontal disease than their non-pregnant peers [5]. Pregnant women with periodontal disease are more susceptible to poor maternal and perinatal outcomes such as preeclampsia [6], gestational diabetes [7], vulvovaginitis, preterm labour, foetal growth restriction [8], low birth weight [9] and perinatal mortality [10].

The prevalence of periodontal disease among pregnant women varies in different populations [11–13]. Moreover, several factors such as age, parity, lower level of education and anaemia were identified as risk factors for periodontal disease during pregnancy [4, 14]. While there is a large amount of data on periodontal disease during pregnancy in other African countries [12, 15, 16], there is no data on periodontal disease during pregnancy in Sudan. There are high rates of maternal and perinatal adverse outcomes in Sudan [17]. Hence, there is a need to investigate the epidemiology of periodontal disease to yield the basic data necessary for the treating physician as well as health planners. The current study was conducted at the antenatal clinic in Saad Abualila Hospital (Khartoum, Sudan) to investigate the prevalence of and risk factors for periodontal disease.

## Main text

### Methods

#### Study design and setting

A cross-sectional study was conducted at the Antenatal Care Clinic of Saad Abualila Hospital (Khartoum, Sudan) from August to October 2018.

#### Section of participants

Pregnant women with singleton pregnancy attending the antenatal care clinic for the first time were approached to be enrolled in the study. Socio-demographic, medical and obstetric history (including history of miscarriage and preterm delivery) was gathered through a questionnaire; then, women were asked specifically about teeth brushing (including number of brushing times) and antibiotic usage during the index pregnancy.

#### Measures

Body mass index (BMI) was computed from the measured weight and height. The dental examination was performed by a single calibrated examiner in the clinic using a dental chair with adequate lighting via an overhead light. Because the participants were pregnant, dental radiographs were not taken. During the examination, probing was performed at six sites per tooth. The pocket depth (the deepest measurement) was identified for each assessed tooth. Pregnant women were diagnosed as having healthy gingiva if no pocketing and no bleeding on probing were noted. We used the minimum diagnostic criteria for periodontal disease in the current study as follows: “bleeding on probing and a pocket depth of at least 4 mm and clinical attachment loss of ≥ 6 mm, calculus with plaque deposits, and gingival recession” [18]. The moderate and severe forms of periodontal disease were defined as the presence of 4 or more sites with a pocket depth > 3 mm and 4 or more sites with pocket depth ≥ 5 mm, respectively. Otherwise, periodontal disease was considered mild.

The sample size was calculated based on a 31.0% prevalence of periodontal disease during pregnancy in a previous study [13]. A total sample size of 404 women was calculated using a single population proportion formula; this sample size will yield a 95% confidence interval, precision of 5.0% and 80% power.

#### Statistical analysis

Data were entered into a computer database and SPSS software (SPSS Inc., Chicago, IL, USA, version 16.0) and double checked before analysis. Means and proportions for the socio-demographic characteristics were compared between the groups of women who had periodontal disease and women who had no periodontal disease using Student’s t test and the χ^2^ test, respectively. Logistic regression analyses were performed using periodontal disease as the dependent variable and socio-demographic characteristics as independent variables. Confidence intervals of 95% were calculated, and *P *< 0.05 was considered significant.

### Results

Four hundreds and four women were enrolled in the study, with a mean (SD) gestational age of 30.0 (8.7) weeks. The mean (SD) age and parity of the investigated women was 27.0 (5.7) years and 1.6 (1.7), respectively. Over one-third (39.0%) of these women had less than a secondary school education level, Table 1.

**Table 1:** Basic socio-demographic characteristics of women (n = 404) who were enrolled in the study

Variable	
Mean (SD) of the	
Age, years	27.0 (5.7)
Parity	1.6 (1.7)
Gestational age, weeks	30.0 (8.7)
Body mass index, kg/m^2^	24.2 (3.8)
Number (proportion) of the	
Educational < secondary level	157 (38.8)
History of miscarriage	85 (21.3)
History of preterm delivery	14 (3.4)
Brushing once	73 (18.1)
Twice	253 (62.6)
More than twice	78 (19.3)
Antibiotic usage	51 (12.6)

Of the 404 women, 73 (18.1%), 253 (62.6%) and 78 (19.3%) were brushing only once, twice and more than twice per day, respectively. Out of these 404 women, 45 (11.1%) had gingival enlargement and only two (0.5%) had pregnancy eruption. Ninety-seven (24.0%) of these 404 women had periodontal disease, which was mild in 49 (12.1%), moderate in 36 (8.9%) and severe in 12 (3.0%). The other 307 (76.0%) had no periodontal disease.

Age, parity, education, history of preterm labour, and brushing were not significantly different between the two groups, but gestational age was significantly lower in women with periodontal disease than in women who had no periodontal disease [27.8 (9.8) vs. 30.7 (8.2), P = 0.004, Table 2].

**Table 2:** Comparing socio-demographic characteristics of women who had periodontal disease and women who had no periodontal disease

Variable	Women who had periodontal disease (n = 97)	Women who had no periodontal disease (n = 307)	P
Age, years	27.1 (5.8)	26.8 (5.6)	0.578
Parity	1.6 (1.7)	1.4 (1.5)	0.200
Gestational age, weeks	27.8 (9.8)	30.7 (8.2)	0.004
Education < secondary level	31 (32.0)	126 (41.0)	0.121
History of miscarriage	23 (23.7)	62 (20.2)	0.476
History of preterm delivery	3 (3.1)	11 (3.6)	0.830
Brushing once	13 (13.4)	60 (19.5)	0.313
Twice	62 (64.0)	191 (62.2)	
More than twice	22 (22.7)	56 (18.2)	
Antibiotic usage	16 (16.5)	35 (11.4)	0.219
Body mass index, kg/m^2^	24.3 (3.5)	24.1 (4.1)	0.067

Likewise, in logistic regression, age, parity, education, and brushing were not associated with periodontitis, but lower gestational age was associated with periodontal disease (OR = 0.96, 95% CI 0.94–0.99, P = 0.011, Table 3).

**Table 3:** Factors associated with periodontal disease according to logistic regression analyses

Variable	OR	95% CI	P
Age, years	0.99	0.94–1.04	0.773
Parity	0.92	0.77–1.10	0.388
Gestational age < 30 weeks	0.96	0.94–0.99	0.011
Educational level < secondary	0.67	0.40–1.13	0.139
History of miscarriage	0.85	0.47–1.52	0.595
History of preterm delivery	0.89	0.62–1.29	0.566
Brushing	1.30	0.88–1.93	0.176
Antibiotic usage	0.64	0.32–1.25	0.195
Body mass index, kg/m^2^	1.00	0.89–1.12	0.953

### Discussion

The main findings of the current study were that there was a high (24.0%) prevalence of periodontal disease among pregnant Sudanese women and that periodontal disease was associated with decrease gestational age. A low (14.2%) prevalence of periodontal disease was recently reported among pregnant Tanzanian women [12]. Likewise, Gomes-Filho el al. reported a low prevalence (17.24%) of periodontal disease among pregnant women in Brazil [19]. A slightly higher prevalence (31%) of periodontal disease was documented in pregnant women in Jordan [13]. Interestingly, Govindasamy et al. recently reported that over half (54.8%) of postpartum women in India had periodontal diseases [11]. Moreover, much higher rates (84.7% and 73.9%) of periodontal disease were reported among women of childbearing age (preconception) in China [20, 21]. Generally, periodontal disease affects between 20% and 50% of pregnant women in industrialized areas [22, 23]. It is worth mentioning that our results should be compared to the results of the latter studies with caution because different methods for diagnosing periodontal disease were followed. Previous studies have revealed that estimates of the prevalence of periodontal conditions and their severity as well as the associated factors vary according to the method used in the particular study [24].

Our study showed no association of age, parity, education or tooth brushing with periodontal disease. In China none of the investigated factors were associated with women of childbearing age, and self-reported bleeding during tooth brushing was the only factor associated with periodontal disease [adjusted odds ratio (aOR): 3.71] [21]. A previous study showed that age (over 30 years) and high parity were associated with periodontal disease during pregnancy [14]. Moreover, higher maternal age and parity were reported to be associated with periodontal disease during pregnancy in Uganda [25]. Moreover, educational level, alcohol consumption, overweight and teeth brushing were associated with periodontal disease in women of childbearing age (preconception) in China [20]. Older age, a lower level of education and unemployment were factors associated with periodontal disease in pregnant women in Bangladesh [4]. The difference between our results and the findings of previous studies could be explained by the different cultures and other socio-demographic variables in the different settings and communities.

The current study found no association between BMI and periodontal disease. Recently, Gomes-Filho el al. reported no association between obesity and periodontal disease among pregnant women in Brazil [19]. On the other hand, Lee et al. reported a significant association between obesity and periodontitis in pregnant women [26]. Although an inflammatory process triggered by a bacterial infection is the main factor involved in periodontal disease, a variety of other factors such as age, nutrition, smoking and genetic susceptibility can influence the progression/severity of the disease [1]. It is worth to be mentioned that our results were mainly on the non- adjusted OR of the logistic regression.

### Conclusions

The current study showed a high prevalence of periodontal disease, which showed no association with, age, parity or BMI. Further research on the maternal and perinatal outcomes and periodontal disease is needed. Moreover the outcome of the treatment of periodontal disease needs to be assessed among pregnant Sudanese women.

## Limitations of the study

We failed to follow up these women; hence, the outcomes of the pregnancies were not known. This was a cross-sectional study and a cause-effect relationship was not determined.

In summary, the current study showed a high prevalence of periodontal disease, which showed no association with, age, parity and BMI.

## Data Availability

The datasets used and/or analysed during the current study are available from the corresponding author on reasonable request.

## Abbreviations

BMI: Body mass index
OR: Odds ratio
SD: Standard deviation

## References

[1] Periodontal Disease Fact Sheet | Perio.org American Academy of Periodontology—Perio.org. IL. Chicago 2017. https://www.perio.org/newsroom/periodontal-disease-fact-sheet. Accessed 4 Dec 2019.

[2] Helmi MF, Huang H, Goodson JM, Hasturk H, Tavares M, Natto ZS (2019). Prevalence of periodontitis and alveolar bone loss in a patient population at Harvard School of Dental Medicine. BMC Oral Health..

[3] Pihlstrom BL, Michalowicz BS, Johnson NW (2005). Periodontal diseases. Lancet..

[4] Anwar N, Zaman N, Nimmi N, Chowdhury TA, Khan MH (2016). Factors associated with periodontal disease in pregnant diabetic women. Mymensingh Med J..

[5] Tettamanti L, Lauritano D, Nardone M, Gargari M, Silvestre-Rangil J, Gavoglio P (2017). Pregnancy and periodontal disease: does exist a two-way relationship?. ORAL Implantol..

[6] Khalighinejad N, Aminoshariae A, Kulild JC, Mickel A (2017). Apical periodontitis, a predictor variable for preeclampsia: a case-control study. J Endod..

[7] Kumar A, Sharma DS, Verma M, Lamba AK, Gupta MM, Sharma S (2018). Association between periodontal disease and gestational diabetes mellitus—a prospective cohort study. J Clin Periodontol.

[8] Figueiredo MGOP, Takita SY, Dourado BMR, de Souza Mendes H, Terakado EO, de Carvalho Nunes HR (2019). Periodontal disease: repercussions in pregnant woman and newborn health—a cohort study. PLoS ONE.

[9] Mathew RJ, Bose A, Prasad JH, Muliyil JP, Singh D (2014). Maternal periodontal disease as a significant risk factor for low birth weight in pregnant women attending a secondary care hospital in South India: a case-control study. Indian J Dent Res.

[10] Bi WG, Emami E, Luo ZC, Santamaria C, Wei SQ (2019). Effect of periodontal treatment in pregnancy on perinatal outcomes: a systematic review and meta-analysis. J Matern-Fetal Neonatal Med.

[11] Govindasamy R, Dhanasekaran M, Varghese S, Balaji V, Karthikeyan B, Christopher A (2017). Maternal risk factors and periodontal disease: a cross-sectional study among postpartum mothers in Tamil Nadu. J Pharm Bioallied Sci..

[12] Gesase N, Miranda-Rius J, Brunet-Llobet L, Lahor-Soler E, Mahande MJ, Masenga G (2018). The association between periodontal disease and adverse pregnancy outcomes in Northern Tanzania: a cross-sectional study. Afr Health Sci..

[13] Alchalabi HA, Al Habashneh R, Jabali Al O, Khader YS (2013). Association between periodontal disease and adverse pregnancy outcomes in a cohort of pregnant women in Jordan. Clin Exp Obstet Gynecol.

[14] Grinin VM, Erkanyan IM, Ivanov SY (2018). Incidence and risk factors of oral diseases in pregnant women. Stomatologiia (Mosk)..

[15] Harjunmaa U, Järnstedt J, Alho L, Dewey KG, Cheung YB, Deitchler M (2015). Association between maternal dental periapical infections and pregnancy outcomes: results from a cross-sectional study in Malawi. Trop Med Int Health..

[16] Muwazi L, Rwenyonyi CM, Nkamba M, Kutesa A, Kagawa M, Mugyenyi G (2014). Periodontal conditions, low birth weight and preterm birth among postpartum mothers in two tertiary health facilities in Uganda. BMC Oral Health.

[17] Ali AA, Okud A, Khojali A, Adam I (2012). High incidence of obstetric complications in Kassala hospital, Eastern Sudan. J Obstet Gynaecol (Lahore).

[18] Armitage GC (2000). Development of a classification system for periodontal diseases and conditions. Northwest Dent..

[19] Gomes-Filho IS, Batista JET, Trindade SC, Passos-Soares JS, Cerqueira EMM, Costa TSD (2020). Obesity and periodontitis are not associated in pregnant women. J Periodontal Res..

[20] Wu YM, Liu J, Sun WL, Chen LL, Chai LG, Xiao X (2013). Periodontal status and associated risk factors among childbearing age women in Cixi City of China. J Zhejiang Univ Sci B..

[21] Jiang H, Su Y, Xiong X, Harville E, Wu H, Jiang Z (2016). Prevalence and risk factors of periodontal disease among pre-conception Chinese women. Reprod Health..

[22] Xiong X, Buekens P, Vastardis S, Yu SM (2007). Periodontal disease and pregnancy outcomes: state-of-the-science. Obstet Gynecol Surv.

[23] Madianos PN, Lieff S, Murtha AP, Boggess KA, Auten RL, Beck JD (2001). Maternal periodontitis and prematurity. Part II: maternal infection and fetal exposure. Ann Periodontol.

[24] Kingman A, Albandar JM (2000). Methodological aspects of epidemiological studies of periodontal diseases. Periodontol.

[25] Wandera M, Astrøm AN, Okullo I, Tumwine JK (2012). Determinants of periodontal health in pregnant women and association with infants’ anthropometric status: a prospective cohort study from Eastern Uganda. BMC Pregnancy Childbirth..

[26] Lee H-J, Jun J-K, Lee S-M, Ha J-E, Paik D-I, Bae K-H (2014). Association between obesity and periodontitis in pregnant females. J Periodontol.

